# Effect of bupivacaine liposome suspension administered as a local anesthetic block on indicators of pain and distress during and after surgical castration in dairy calves

**DOI:** 10.1093/jas/skab378

**Published:** 2021-12-30

**Authors:** Miriam S Martin, Michael D Kleinhenz, Abbie V Viscardi, Andrew K Curtis, Blaine T Johnson, Shawnee R Montgomery, Maria E Lou, Johann F Coetzee

**Affiliations:** 1 Department of Anatomy and Physiology, Kansas State University College of Veterinary Medicine, Manhattan, KS 66506, USA; 2 Department of Clinical Sciences, Kansas State University College of Veterinary Medicine, Manhattan, KS 66506, USA; 3 Department of Diagnostic Medicine and Pathobiology, Kansas State University College of Veterinary Medicine, Manhattan, KS 66506, USA

**Keywords:** analgesia, castration, cattle, pain

## Abstract

Castration is a routine procedure performed on beef and dairy operations in the United States. All methods of castration cause behavioral, physiologic, and neuroendocrine changes associated with pain. The American Veterinary Medical Association and the American Association of Bovine Practitioners recommend that anesthesia and analgesia be administered during castration. The objective of this study was to evaluate the effectiveness of bupivacaine liposome suspension, a novel, long-acting, local anesthetic formulation administered as a nerve block at castration. The authors chose to investigate this novel formulation as an alternative to the current industry standards using lidocaine nerve blocks alone or in combination with meloxicam. Thirty male Holstein calves, 16 to 20 wk of age, were enrolled and randomly assigned to one of the four treatment groups prior to surgical castration: 1) bupivacaine liposome suspension block + oral placebo (**BUP**), 2) lidocaine block + oral placebo (**LID**), 3) lidocaine block + oral meloxicam (1 mg/kg) (**LID + MEL**), and 4) saline block + oral placebo (**CON**). Biomarkers were collected at −24 h and from 0 to 120 h post-castration and included infrared thermography, pressure mat gait analysis, chute defense and behavior scoring (pain and activity), and blood sampling for serum cortisol and prostaglandin E_2_ metabolites (**PGEM**s). Responses were analyzed using repeated measures, with calf nested in treatment as a random effect, and treatment, time, and their interaction designated as fixed effects. The results from pressure mat gait analysis show that the CON had a shorter front limb stance time from baseline (−8.73%; 95% confidence interval [**CI**]: −24.84% to 7.37%) compared with BUP and LID + MEL (>5.70%; 95% CI: −22.91% to 23.79%) (*P* < 0.03). The CON tended to have an increase in front limb force from baseline (6.31%; 95% CI: −1.79% to 14.41%) compared with BUP, LID, and LID + MEL (<−5.06%; 95% CI: −14.22% to 0.95%) (*P* < 0.04). The CON displayed higher counts of hunched standing (2.00; 95% CI: 1.68 to 2.32) compared with LID + MEL (1.43; 95% CI: 1.13 to 1.72) (*P* = 0.05). The CON had higher cortisol concentrations at 24 h (7.70 ng/mL; 95% CI: 1.52 to 13.87 ng/mL) relative to BUP (3.11 ng/mL; 95% CI: −2.56 to 8.79 ng/mL) (*P* = 0.002). At 4 and 24 h, LID + MEL had lower PGEM concentrations from baseline (−32.42% and −47.84%; 95% CI: −78.45% to −1.80%) compared with CON (27.86% and 47.63%; 95% CI: 7.49% to 82.98%) (*P* < 0.02). The administration of bupivacaine liposome suspension as a local anesthetic block at the time of castration was as effective at controlling pain as a multimodal approach of lidocaine and meloxicam.

## Lay Summary

Castration is a routine procedure performed on beef and dairy operations in the United States. All methods of castration cause pain. The American Veterinary Medical Association and the American Association of Bovine Practitioners recommend that anesthesia and analgesia be administered during castration. The objective of this study was to evaluate the effectiveness of bupivacaine liposome suspension, a novel, long-acting, local anesthetic formulation administered as a nerve block at castration, as an alternative to current industry standards using lidocaine nerve blocks alone or in combination with meloxicam. Evidence provided in the current study demonstrates that pain from surgical castration can last up to 120 h post-castration, indicated by changes in ocular temperature, gait analysis, and prostaglandin metabolite concentrations. These data show that the administration of bupivacaine liposome suspension as a local anesthetic block at the time of castration was as effective at controlling pain as a multimodal approach of lidocaine and meloxicam. A single injection that alleviates both perioperative and postoperative pain would be an attractive option for livestock producers to alleviate pain at the time of castration. Further research is needed to discover effective ways of managing pain for extended durations following painful husbandry procedures.

## Introduction

Castration is routinely performed on beef and dairy operations in the United States. Dairy calves are castrated, on average, at 7.5 wk, and the majority of beef calves are castrated prior to being sold off the ranch (USDA-APHIS-NAHMS, 2018, 2020). Surgical castration methods are the most common method in the United States. (Coetzee et al., 2008). All castration methods cause behavioral, physiologic, and neuroendocrine changes associated with pain (Coetzee, 2011). Wound healing ranges from 35 to 56 d following surgical castration (Marti et al., 2018).

The American Veterinary Medical Association and the American Association of Bovine Practitioners recommend that anesthesia and analgesia be administered during castration (AVMA, 2014; AABP, 2019). Yet, less than 25% of producers report always using local anesthesia or analgesia for surgical castration in calves < 12 mo of age (Johnstone et al., 2021). One factor contributing to the low adoption rate is the lack of Food and Drug Administration (FDA)-approved analgesics (Robles et al., 2021). There are no analgesics approved to control pain associated with castration in food animals in the United States. However, veterinarians are permitted to prescribe analgesics for extra-label purposes under the Animal Medicinal Drug Use Clarification Act (**AMDUCA**; FDA, 1994). Lidocaine, the original amino-amide local anesthetic that is most widely used in veterinary practice, has a limited duration of action, and bupivacaine, which is among the most potent and long acting amino amides, has a longer duration of action but is thought to have a later onset (Best et al., 2015). 

A liposomal formulation of bupivacaine was approved for dogs in 2016 that provides up to 72 h of pain control (FDA, 2016). Liposomal bupivacaine has an increased duration of action and a delayed peak plasma concentration when compared with bupivacaine hydrochloride (Tong et al., 2014). Meloxicam is a nonsteroidal anti-inflammatory drug (**NSAID**) with preferential cyclooxygenase-2 activity (Smith, 2013), which is a practical analgesic option for producers due to its half-life of 27 h (Coetzee et al., 2009). Meloxicam has been shown to reduce pain responses up to 72 h after castration (Olson et al., 2016). The objective of this study was to evaluate the effectiveness of bupivacaine liposome suspension, a novel, long-acting, local anesthetic formulation administered as a nerve block during castration. The authors chose to investigate this novel formulation as an alternative to the current industry standards using lidocaine nerve blocks alone or in combination with meloxicam. The null hypothesis was that there would be no difference in analgesic effectiveness in controlling pain at the time of castration.

## Materials and Methods

### Animals, housing, and treatments

The Kansas State University Institutional Animal Care and Use Committee (**IACUC**) reviewed and approved the experimental protocol for this project (IACUC# 4445). This study was conducted at the Kansas State University College of Veterinary Medicine in Manhattan, KS, in September 2020. All calves were assessed three times daily for signs of excessive pain via behavior and inappetence for a 120-h period after castration, in consideration for calves in the control group experiencing pain associated with castration. The administration of flunixin meglumine (2.2 mg/kg, IV, q 12 h) was established as the rescue analgesic protocol for calves showing excessive lying, reluctance to rise, or inappetence following castration. A different analgesic was chosen for the rescue protocol in the case that the calf may have already received meloxicam. A total of 30 weaned, vaccinated, and intact male Holstein calves were received from a producer for potential enrollment onto the study in June 2020.

Calves were group housed in two outdoor pens with open front run-in sheds of equal size for shelter. Pens were long runs where calves could easily disperse with concrete flooring and size exceeding the guidelines for calf housing in the *Guide for Care and Use of Agricultural Animals in Research and Teaching* (FASS, 2020). Calves were fed a grain diet formulated at 3.5% BW twice daily per normal procedures at the study site along with free-choice hay. Calves were moved to the study site upon arrival and were given a 2-wk acclimation period; throughout the acclimation period, calves were trained to be led with a halter and stand haltered for an extended period of time to facilitate biomarker collection.

After the acclimation period, prior to the start of the study, calves were weighed and averaged 155 kg (range: 121 to 187 kg). Calves (*n* = 30) were 16 to 20 wk of age at the time of enrollment. Calves were castrated in two groups of 15 and were randomly allocated to a castration group, as well as one of the four experimental treatment groups. The first 15 calves castrated were housed in a single pen which included calves from each of the four experimental treatment groups. The second 15 calves castrated were housed in a single pen which included calves from each of the four experimental treatment groups. Calves were randomized by weight. Randomization was accomplished using the RAND function in Microsoft Excel (Microsoft Excel 2016, Microsoft Corporation, Redmond, WA). The treatment groups were as follows: 1) bupivacaine liposome suspension block + oral placebo (**BUP**), 2) lidocaine block + oral placebo (**LID**), 3) lidocaine block + oral meloxicam (1 mg/kg) (**LID + MEL**), and 4) saline block + oral placebo (**CON**). The oral meloxicam tablets (Zydus Pharmaceuticals Inc., Pennington, NJ) were placed in a gelatin capsule (Torpac Inc., Fairfield, NJ) and were administered via a bolus gun. As with the oral meloxicam tablets, the oral placebo was also placed into a gelatin capsule (Torpac Inc.) and administered via a bolus gun. The oral placebo formulation was lactose monohydrate (Thermo Fisher Scientific, Waltham, MA) which is the binder used in meloxicam tablets. Eight calves were assigned to each of the BUP, LID, and LID + MEL treatment groups, and six calves were assigned to the CON treatment group. Each treatment was equally represented in each castration group. The calf was the experimental unit for the study. The duration of outcome variable collection was 120 h post-castration, with baseline measures collected 24 h prior to calves being castrated. The time of castration was considered the 0 h time point.

Treatments were administered 10 min prior to the castration procedure. The scrotum and testicles were anesthetized by injecting 2 mL of lidocaine (MWI, Boise, ID) or bupivacaine liposome suspension (NOCITA, Elanco US Inc., Greenfield, IN) using a 20-gauge needle into the neck of the scrotum (proximal scrotum) and 3 to 4 mL around each spermatic cord (8to 10 mL total lidocaine or bupivacaine liposome suspension). Ten minutes following the local anesthetic block, calves were surgically castrated as described: the scrotum was cleaned and disinfected by applying a mix of water and chlorhexidine and using sterile gauze until there was no visible debris, the distal half of the scrotum was surgically incised using a disposable scalpel blade, the testes and spermatic cord were exteriorized by blunt dissection, and the testicles were removed by stripping and twisting the spermatic cord.

### Measurements and sample collection

**Figure 1.**
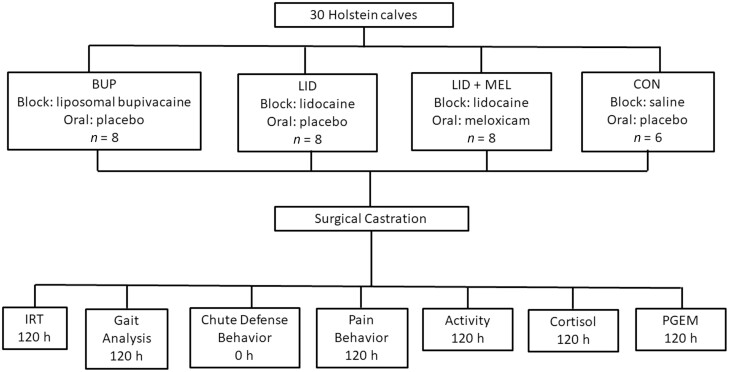
Flow chart outlining the timing of study events. Calves were castrated and outcome variables were collected at baseline and for the duration of time expressed in h (0 to 120) below each specific outcome variable.

Outcome variables were collected at −24, 0, 0.5, 1, 2, 4, 8, 24, 72, and 120 h post-treatment, with chute defense behavior being scored at 0 h (at the time of castration) and pressure mat gait analysis beginning at 4 h and being collected for all of the remaining time points. Outcome variables collected included: infrared thermography (**IRT**), gait analysis using a pressure mat, chute defense and behavior scoring (pain and activity), and blood sampling for serum cortisol and prostaglandin E_2_ metabolites (**PGEM**s) (**Figure 1**). All trained evaluators were masked to treatment for the duration of the study. A veterinarian (M.D.K.) administered the treatments and was the only one with access to which calves received which treatment. The trained evaluators could then remain masked for the study duration for sample collection as well as during data analysis.

### Infrared thermography

A research-grade infrared camera (Fluke TiX580, Fluke Corp, Everett, WA) was used to capture IRT images of the medial canthus of the left eye applying methods adapted from Kleinhenz et al. (2017). Images were obtained 0.5 m from the left eye of the calf. Infrared images were analyzed using research-specific computer software (SmartView v. 4.3, Fluke Thermography, Plymouth, MN) to determine maximum and minimum temperatures.

### Pressure mat gait analysis

A commercially available pressure mat gait analysis system (Walkway, Tekscan, Inc., South Boston, MA) was used to record gait and biomechanical parameters. The pressure mat was calibrated, using a known mass, to ensure accuracy of measurements at each time point. Video synchronization (Logitech, Newark, CA) was used to ensure consistent gait between and within calves at each time point. Using research-specific software (Walkway 7.7, Tekscan, Inc.), force, contact pressure, impulse, stance time, stride length, velocity, and gait distance were assessed. A percent change from baseline was calculated for each output and used for statistical analysis.

### Behavior

Video cameras (Sony Handycam HDR-CX405, Sony Corporation of America, New York, NY) were placed on tripods outside of the chute area for the 0 and 1 h time points or calf pens for the 2 to 24 h time points, based on when calves returned to their home pen following castration.

Chute defense behavior was scored at the time of castration using the scale adapted from Grandin (1993) and also cited by Hoppe et al. (2010), summarized as 1) calm, no movement; 2) restless, shifting; 3) squirming, occasionally shaking of the chute; 4) continuous vigorous movement and shaking of the chute; and 5) rearing, twisting of the body, or violent struggling.

A pain score was also assigned at the time of castration that was adapted from the pain scale developed by Gleerup et al. (2015) (**Table 1**). The behaviors summed in the pain score were back position, head position, ear position, and facial expression along with an additional variable the authors chose to include—vocalization. Back position, head position, and ear flicking were also evaluated as stand-alone pain behaviors.

**Table 1.** Modified pain scale used to score calf behavior and assign a pain score (adapted from Gleerup et al., 2015)

Score	0	1	2
Head position	High/level of withers Animal is active, eating, ruminating, or contact seeking/curious	Level of withers Animal is not active, not eating, ruminating, grooming, or sleeping	Low Animal is not active, not eating, ruminating, grooming, or sleeping; may lie down quickly after getting up
Ear position	Both ears forward or one ear forward or back and the other listening	Ears back/asymmetric ear movements Both ears back or moving in different directions	Both ears to the sides and lower than usual (i.e., lambs’ ears); the pinna facing slightly down
Facial expression	Attentive/neutral look Animal is attentive, focused on a task (e.g., eating or ruminating), or sleeping	Tense expression/strained appearance Animal has a worried or strained look, furrows above the eyes, and puckers above the nostrils	
Back position	Normal	Slightly arched back	Arched back
Vocalization	No vocalization	Vocalization	

Calves were video recorded the day prior to the castration procedure for 30 min, to collect baseline pain behavior and activity data. Post-castration, calves were video recorded for 30 min at the following time points: 0, 1, 2, 4, 8, and 24 h. The videos were randomized across time point and calf ID using a random number generator (random.org). Three trained observers blinded to treatment and time point used a detailed ethogram (**Table 2**) and BORIS software (Behavioral Observation Research Interactive Software v 7.7.3, Torino, Italy) to score calf behavior. Focal-animal, continuous sampling was used for behavioral analysis (footage was watched in its entirety to record one focal animal’s behavior and then was re-watched to score the next focal animal). Pain behaviors (attention to the surgical site, licking the surgical site, tail flicking, and foot stamping) were classified as events, and the occurrence of each behavior (i.e., count data) was collected. The rest of the behaviors in the ethogram were classified as states, and the total duration(s) of these behaviors across the observation period was collected. A total of 6,300 min (105 h) of behavior recordings were scored and analyzed for this study. The inter-observer reliability between the three individuals scoring behavior was assessed by having all observers score the same calf in three different videos for 30 min and then calculating the interclass correlation coefficient (**ICC**). The ICC was ≤0.9 between the three individuals scoring behavior across three different samples of video footage, indicating excellent reliability between observers.

**Table 2.** Ethogram used to score calf behavior and activity (adapted from Heinrich et al., 2010 and Sutherland et al., 2013)

Behavior	Description
Eating	Ingesting food provided at feed bunk
Drinking	Consuming water from a bucket or waterer
Ruminating	Regurgitating, chewing, and swallowing food
Grooming	Calf moves the tongue over body, licking
Walking	Moving forward at a normal pace
Standing	Calf is upright and all four hooves are in contact with the ground
Lying	Calf is recumbent; the body is in contact with the ground
Attention to surgical site	Turning head back toward hind end with attention focused on the scrotal area. May involve lifting a hind limb. No attempts are made to lick the surgical site
Licking/attempting to lick surgical site	Lifting a hind limb and licking (or attempting to lick) the scrotal area
Tail flicking	Calf rapidly moves tail from side to side. May include multiple tail movements within one tail-flicking event. A new tail flicking event occurs after the tail moves slowly or is in a resting position
Foot stamping	Calf raises one foot and brings it down again firmly

### Blood sampling

Blood samples for serum cortisol and PGEM determination were collected from the jugular vein via venipuncture. The whole blood samples were immediately transferred to tubes (Vacutainer, BD Diagnostics, Franklin Lakes, NJ) containing either no additive for cortisol determination or ethylenediaminetetraacetic acid (EDTA) anticoagulant for PGEM determination. Blood samples were then centrifuged for 10 min at 1,500 × *g*; serum and plasma were placed in cryovials via transfer pipette and stored at −80 °C.

### Cortisol

Serum cortisol concentrations were determined using a commercially available radioimmunoassay kit (MP Biomedicals, Irvine, CA) following the manufacturer’s specifications with minor modifications as described in Martin et al. (2021). The standard curve was extended to include 1 and 3 ng/mL by diluting the 10 and 30 ng/mL manufacturer-supplied standards 1:10, respectively. The standard curve ranged from 1 to 300 ng/mL. A low (25 ng/mL) and high (150 ng/mL) quality controls (**QCs**) were run at the beginning and end of each set to determine inter-assay variability. Plain 12 × 75-mm polypropylene tubes were used as blank tubes to calculate nonspecific binding. Input for standards, QCs, and samples were adjusted to 50 µL. Samples were incubated at room temperature for 30 min prior to the addition of I-125. Manufacturer instructions were then followed. Tubes were counted on a gamma counter (Wizard2, PerkinElmer, Waltham, MA) for 1 min. The raw data file was then uploaded onto MyAssays Desktop software (version 7.0.211.1238, 21 Hampton Place, Brighton, UK) for concentration determination. Standard curves were plotted as a 4-parameter logistic curve. Samples with a coefficient of variation (CV) ˃ 18% were re-analyzed. The projected average for serum cortisol intra-assay CV was 18.9% and inter-assay CV was 20.81%, with the average low-QC CV being 20.15% and average high-QC CV being 20.86%.

### Prostaglandin E_2_ metabolites

PGEMs were analyzed using a commercially available ELISA kit (cat. no. 514531, Cayman Chemical, Ann Arbor, MI) following the manufacturer’s specifications with minor modifications. Sample input was adjusted to 375 µL with 1.5 mL ice-cold acetone added for sample purification. Samples were incubated at −20 °C for 30 min and then centrifuged at 3,000 × *g* for 5 min. The supernatant was transferred to clean 13 × 100-mm glass tubes and evaporated using a CentriVap Concentrator (cat. no. 7810014, Labconco, Kansas City, MO) overnight (approx. 18 h). Samples were reconstituted with 375 µL of appropriate kit buffer. A 300-µL aliquot of the reconstituted sample was derivatized with proportionally adjusted kit components. Manufacturer protocol was then followed. Samples were diluted at 1:2 and ran in duplicate. Absorbance was measured at 405 nm after 60 min of development (SpectraMax i3, Molecular Devices, San Jose, CA). Sample results were excluded if the raw read exceeded the raw read of the highest standard (Standard 1; 50 pg/mL) or was below the lowest acceptable standard. The lowest acceptable standard was defined for each individual plate and was identified by excluding standards that had a ratio of absorbance of that standard to the maximum binding of any well (%B/B_0_) of ≥80% or ≤20%. Any individual sample outside the standard curve, with a %B/B_0_ outside the 20% to 80% range, or a CV ˃ 15% was re-analyzed. The projected average for PGEM intra-assay CV was 15.08% and inter-assay CV was 11.40%.

### Calculations and statistical analysis

A sample size calculation was performed a priori using serum cortisol means derived from Martin et al. (2021) to determine a sample size of seven calves per treatment group. The study was designed to have power exceeding 0.80 assuming a difference in effect size (Δ) of 1.2 ng/mL, a standard error (σ) of 0.8, and a statistical inference level (α) of 0.05. Concentrations of serum cortisol and PGEM were log-transformed for normality before the statistical analysis. Responses (i.e., IRT, gait analysis, behavior, serum cortisol, and PGEM) were analyzed using repeated measures with the calf as the experimental unit. Calves nested in a treatment group were designated as a random effect, with treatment, time, and treatment by time interaction designated as fixed effects. *F* tests were utilized for testing the significance of main effects and interactions. If significant overall differences were identified, pairwise comparisons were performed using the Tukey honestly significant difference test. Statistics were performed using statistical software (JMP Pro 15.1.0 and Statistical Analysis System 9.4, SAS Institute, Inc., Cary, NC). Statistical significance was set a priori at *P* ≤ 0.05 with *P* ≤ 0.10 considered to be a trend toward significance. Data are presented as least squares means.

## Results

None of the calves required rescue analgesia. We found no evidence of a treatment effect for IRT maximum ocular temperature (*P* = 0.80). Treatment groups had similar maximum ocular temperatures. There was a significant time effect (*P* < 0.01) with the highest ocular temperatures at 72 and 120 h (37.16 °C; 95% confidence interval [**CI**]: 34.65 to 39.66 and 37.01 °C; 95% CI: 34.51 to 39.52 °C, respectively) relative to all other time points (<36.7 °C) (*P* < 0.05).

**Figure 2.**
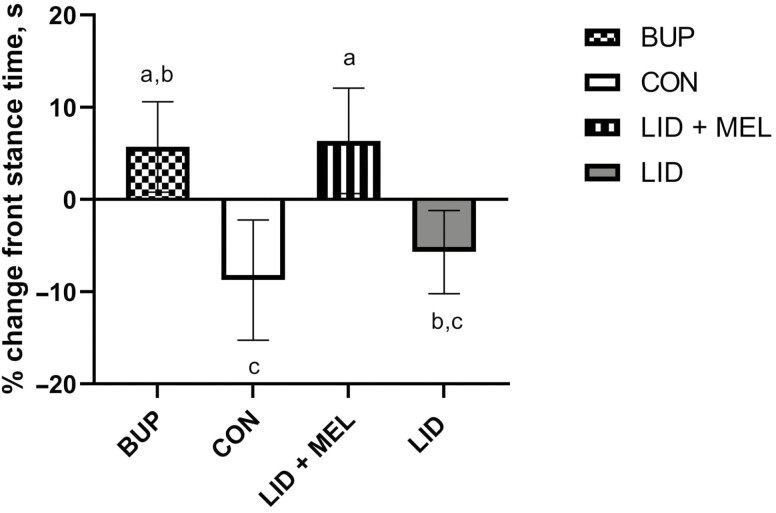
Mean percent change from baseline for front stance time (s) over the duration of the study for each of the four treatment groups. Error bars indicate SEM. Different superscripts (^a,b,c^) indicate significant differences between time points (*P* ≤ 0.05). Treatments: BUP, bupivacaine liposome suspension block + oral placebo; CON, saline block + oral placebo; LID + MEL, lidocaine block + oral meloxicam (1 mg/kg); LID, lidocaine block + oral placebo.

The percent change in front stance time(s) differed significantly between treatment groups (*P* = 0.04) with the LID + MEL group (6.35%: 95% CI: −11.77% to 24.48%) having a more positive percent change from the LID and CON groups (<−5.70%; 95% CI: −24.84% to 11.51%) (*P* < 0.05) and the CON group (−8.73%: 95% CI: −24.84% to 7.37%) having a more negative percent change from the BUP and LID + MEL groups (>5.70%; 95% CI: −22.91% to 23.79%; *P* < 0.03; **Figure 2**). The percent change in front force (kg) between treatment groups trended toward significance (*P* = 0.06). The CON group (6.31%: 95% CI: −1.79% to 14.41%) had a more positive percent change in force compared with the BUP, LID, and LID + MEL groups, which showed negative changes (<−5.06%; 95% CI: −14.22% to 0.95%; *P* < 0.04). There was evidence of a trend toward significance for a treatment by time interaction for front pressure (kg/cm^2^) (*P* = 0.07). At the 2-h time point, the LID group had a more positive percent change in front pressure (7.65%; 95% CI: −5.62% to 20.92%) relative to the CON group (−16.30%; 95% CI: −32.37% to −0.24%; *P* = 0.02). There was no evidence of a treatment effect for percent change in gait distance (cm) (*P* = 0.17), velocity (cm/s) (*P* = 0.46), front stride length (cm) (*P* = 0.22), front impulse (kg ∗ s) (*P* = 0.38), rear stance time (s) (*P* = 0.35), rear stride length (cm) (*P* = 0.14), rear force (kg) (*P* = 0.54), rear impulse (kg ∗ s) (*P* = 0.37), or rear pressure (kg/cm^2^) (*P* = 0.99).

**Figure 3.**
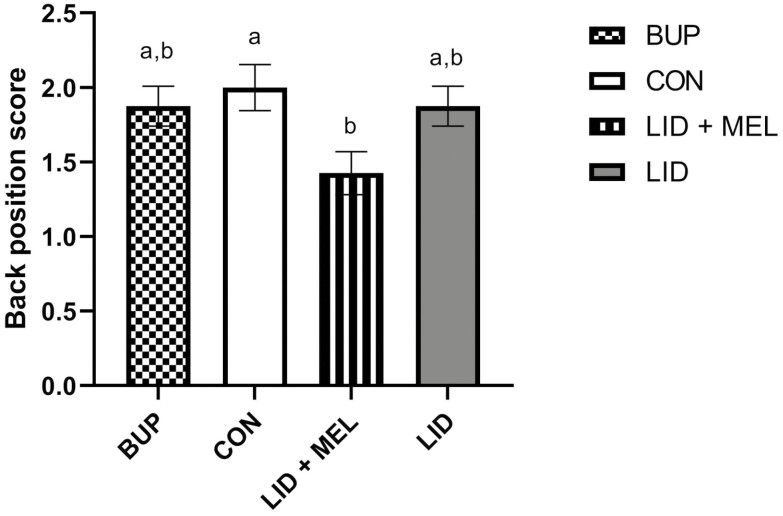
Mean back position score (0 to 2) during castration for each of the four treatment groups. Error bars indicate SEM. Different superscripts (^a,b^) indicate significant differences between time points (*P* ≤ 0.05). Treatments: BUP, bupivacaine liposome suspension block + oral placebo; CON, saline block + oral placebo; LID + MEL, lidocaine block + oral meloxicam (1 mg/kg); LID, lidocaine block + oral placebo.

There was no evidence of a treatment effect for chute defense behavior (*P* = 0.58). There was no evidence of a treatment effect for pain score (*P* = 0.11). However, the back position did differ by treatment (*P* = 0.05; **Figure 3**). The LID + MEL group had a significantly lower back position score (1.43; 95% CI: 1.13 to 1.72) compared with the CON group (CON: 2.00; 95% CI: 1.68 to 2.32) (*P* = 0.05). There was no evidence of treatment effect for ear position or head position (*P* = 0.59 and *P* = 0.49, respectively).

**Figure 4.**
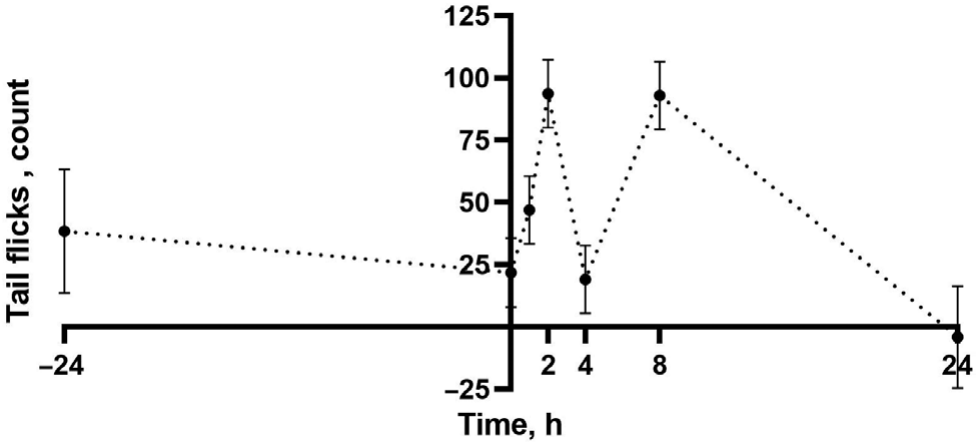
Mean tail flicks (count) over the first 24 h of the study. Error bars indicate SEM.

**Figure 5.**
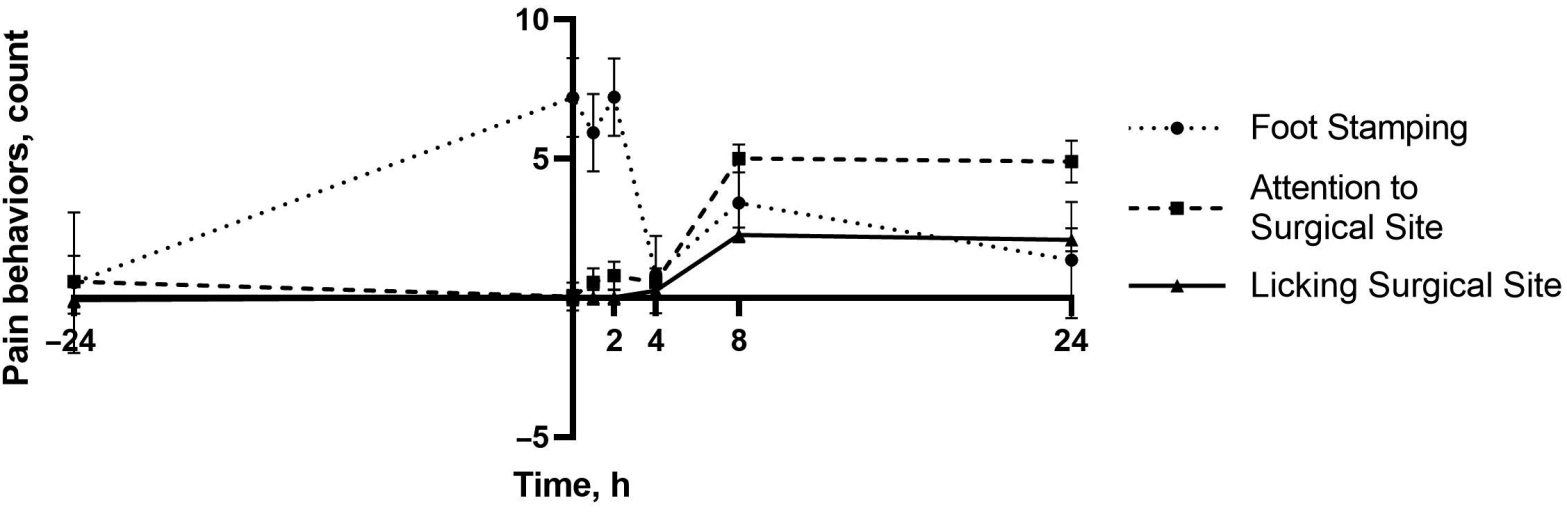
Mean pain behaviors (count) including attention to surgical site, foot stamping, and licking the surgical site over the first 24 h of the study. Error bars indicate SEM.

Tail flicking differed by time (*P* < 0.01) with more tail flicking at 2 and 8 h (93.72 and 92.96 times; 95% CI: 66.7 to 120.73 and 65.94 to 119.97 times, respectively) compared with 0 and 24 h (21.86 and −4.10 times; 95% CI: −5.63 to 49.34 and −44.72 to 36.53 times, respectively; *P* < 0.01; **Figure 4**). Foot stamping differed by time (*P* = 0.01) with more foot stamping at 0 and 2 h (7.19 and 7.21 times; 95% CI: 4.40 to 9.98 and 4.47 to 9.95 times, respectively) compared with 4 h (0.83 times; 95% CI: −1.91 to 3.57 times; *P* < 0.05; **Figure 5**). There were trends toward significance by treatment for foot stamping (*P* = 0.10) and attention to the surgical site (*P* = 0.10) with the CON group stamping the least (1.58 times; 95% CI: −0.96 to 4.13 times) and paying the most attention to the surgical site (2.44 times; 95% CI: 1.52 to 3.36 times). Attention to surgical site differed by time, with the most attention paid to the surgical site at 8 and 24 h (5.01 and 4.89 times; 95% CI: 4.01 to 5.99 and 3.39 to 6.38 times, respectively) relative to all other time points (<0.57 times; 95% CI: −0.97 to 1.87 times; *P* < 0.01; **Figure 5**). Licking the surgical site differed by time at 8 and 24 h (2.26 and 2.08 times; 95% CI: 1.73 to 2.80 and 1.27 to 2.88 times, respectively) compared with all other time points (<0.27 times; 95% CI: −0.53 to 0.90 times) as well (*P* < 0.02; **Figure 5**).

Eating differed by time (*P* < 0.01) with less time spent eating at 4 and 8 h (225 and 207 s; 95% CI: 77.33 to 373.02 and 42.87 to 372.54 s, respectively) relative to −24 and 24 h (974 and 869 s; 95% CI: 731.90 to 1,217.01 and 644.43 to 1,093.94 s, respectively; *P* < 0.01). There was a trend toward significance (*P ≤* 0.10) by treatment for ruminating (*P* = 0.06) with calves in the CON group spending the most time ruminating (568 s; 95% CI: 256.80 to 880.12 s; *P* = 0.06). Standing differed by time (*P* < 0.01) with less time spent standing at 4 h (375 s; 95% CI: 231.25 to 520.56 s) compared with all other time points (<1,319 s; 95% CI: 1,141.25 to 2,000.76 s; *P* < 0.01). Walking differed by time (*P* < 0.01) with more time spent walking at −24, 2, and 8 h (127, 127, and 113 s; 95% CI: 79.94 to 175.26, 101.54 to 153.82, and 86.29 to 141.55 s, respectively) compared with all other time points (<50 s; 95% CI: −12.27 to 77.01 s; *P* < 0.02). There was no evidence of a treatment or time effect for drinking, grooming, or lying behavior (*P* > 0.20).

**Figure 6.**
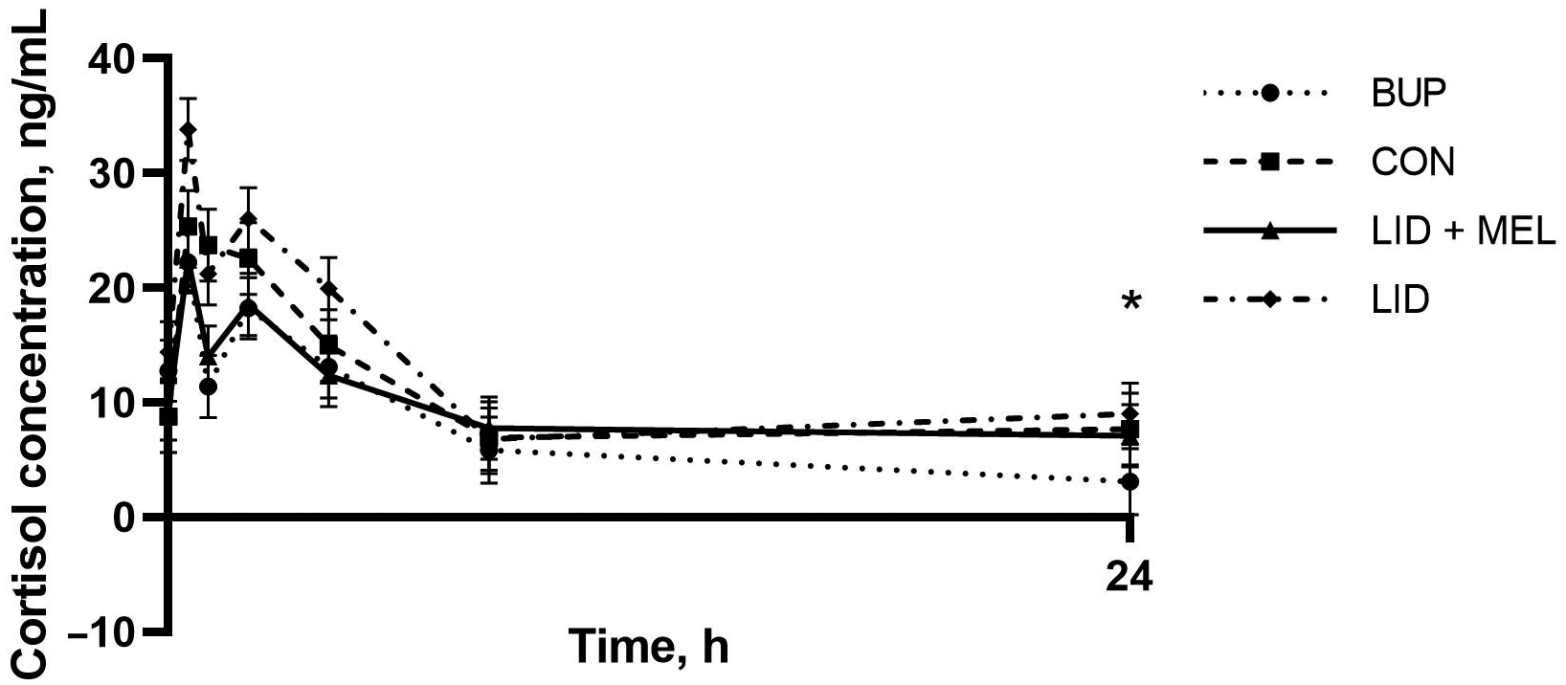
Mean cortisol concentration (ng/mL) for the first 24 h of the study for each of the four treatment groups. Error bars indicate SEM. ∗Denotes time points where a statistically significant difference (*P* ≤ 0.05) was observed between at least two treatment groups. Treatments: BUP, bupivacaine liposome suspension block + oral placebo; CON, saline block + oral placebo; LID + MEL, lidocaine block + oral meloxicam (1 mg/kg); LID, lidocaine block + oral placebo.

There was evidence of a trend toward significance (*P *≤ 0.10) for a treatment by time interaction for average cortisol concentrations (ng/mL) when log transformed (*P* = 0.12; **Figure 6**). Calves in the CON group had lower cortisol concentrations at −24 h (2.41 ng/mL; 95% CI: −4.32 to 9.14 ng/mL) relative to 0, 0.5, 1, 2, 4, 8, and 24 h (≥6.95 ng/mL: 95% CI: 0.78 to 29.91 ng/mL; *P* < 0.01). Calves in the LID group had lower cortisol concentrations at −24 h (5.85 ng/mL; 95% CI: −0.28 to 11.98 ng/mL) relative to 0, 0.5, 1, 2, and 4 h (≥14.41 ng/mL: 95% CI: 9.09 to 39.13 ng/mL; *P* < 0.01). Calves in the LID + MEL group had lower cortisol concentrations at −24 h (4.01 ng/mL; 95% CI: −2.11 to 10.14 ng/mL) relative to 0, 0.5, 1, 2, and 4 h (≥9.42 ng/mL: 95% CI: 4.10 to 27.79 ng/mL; *P* < 0.04). Calves in the BUP group had lower cortisol concentrations at −24 h (2.77 ng/mL; 95% CI: −4.71 to 10.26 ng/mL) relative to 0, 0.5, 1, 2, and 4 h (≥11.40 ng/mL: 95% CI: 6.09 to 27.53 ng/mL; *P* < 0.01). Calves in the CON group had higher cortisol concentrations at 24 h (7.70 ng/mL; 95% CI: 1.52 to 13.87 ng/mL) relative to the BUP group (3.11 ng/mL; 95% CI: −2.56 to 8.79 ng/mL; *P* = 0.02). Calves in the CON group had lower cortisol concentrations at 120 h (1.82 ng/mL; 95% CI: −6.79 to 10.43 ng/mL) relative to the LID + MEL group (8.34 ng/mL; 95% CI: 0.92 to 15.76 ng/mL; *P* = 0.01).

**Figure 7.**
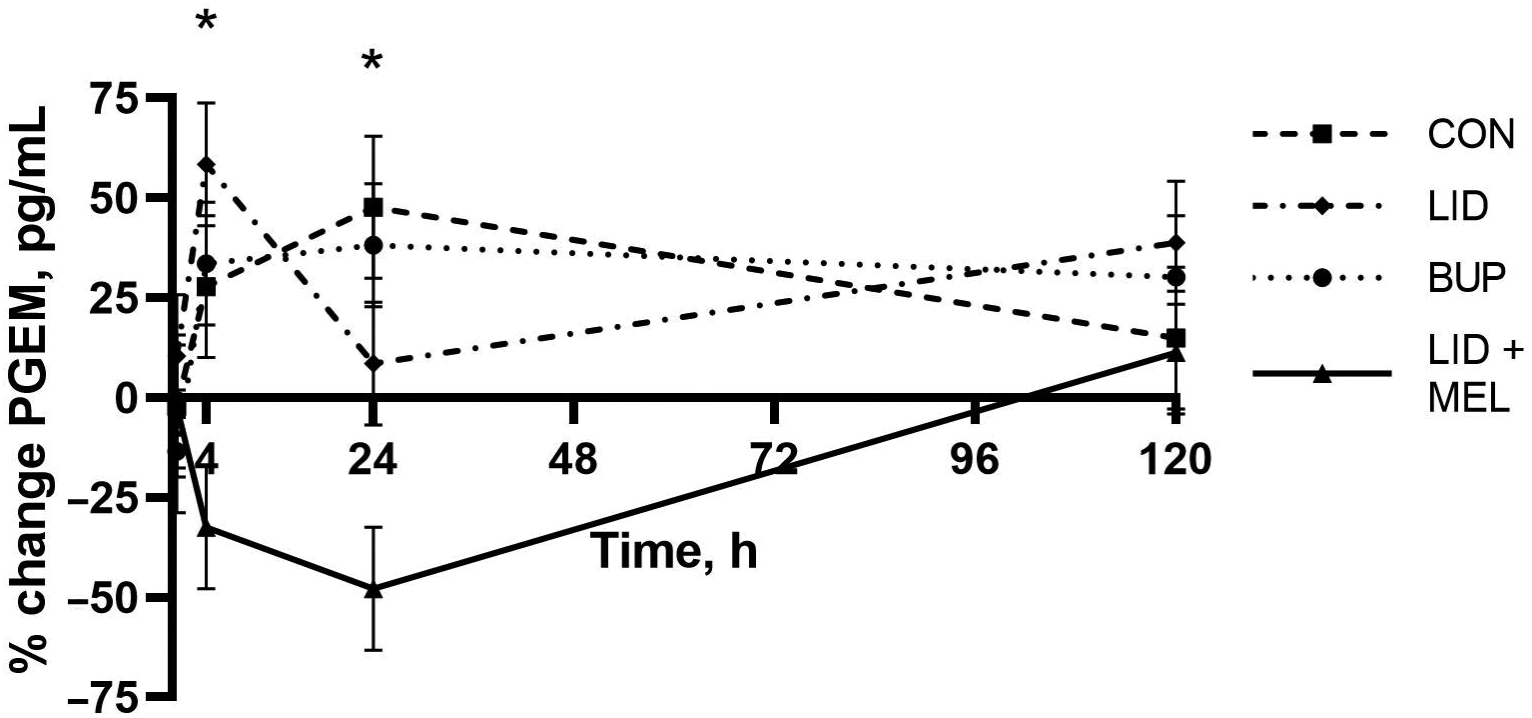
Mean percent change from baseline for prostaglandin E_2_ metabolite (PGEM) concentration (pg/mL) over the duration of the study for each of the four treatment groups. Error bars indicate SEM. ∗Denotes time points where a statistically significant difference (*P* ≤ 0.05) was observed between at least two treatment groups. Treatments: BUP, bupivacaine liposome suspension block + oral placebo; CON, saline block + oral placebo; LID + MEL, lidocaine block + oral meloxicam (1 mg/kg); LID, lidocaine block + oral placebo.

There was evidence of a significant treatment by time interaction for percent change from baseline in PGEM concentration (pg/mL) (*P* = 0.01; **Figure 7**). At 4 h, the LID + MEL group had a negative percent change from baseline (−32.42%: 95% CI: −63.03% to −1.80%) that differed significantly from the positive percent change in the CON group (27.86%: 95% CI: −7.49% to 63.20%; *P* = 0.01). At 24 h, the LID + MEL group had a negative percent change from baseline (−47.84%: 95% CI: −78.45% to −17.23%) that differed significantly from the positive percent change in the CON group (47.63%: 95% CI: 12.28% to 82.98%; *P* = 0.01).

## Discussion

Lidocaine is a widely used local anesthetic in veterinary practice which is likely attributable to its low cost and long shelf-life (Riviere and Papich, 2018). A multimodal approach of lidocaine and meloxicam has been well characterized as a more effective option for controlling pain associated with painful husbandry procedures, due to a longer duration of action than lidocaine alone and lidocaine and meloxicam in reducing pain at different time points (Heinrich et al., 2010; Allen et al., 2013; Laurence et al., 2016; Meléndez et al., 2018b). Bupivacaine liposome suspension is a novel option for pain control during castration. Based on the results of the present study, the bupivacaine liposome suspension did not have a delayed onset relative to lidocaine, and it was able to control pain for a duration similar to a multimodal approach of lidocaine and meloxicam. In a recent study comparing the effect of bupivacaine liposome suspension to a combination of lidocaine and meloxicam on pain biomarkers at the time of dehorning, similar results were observed for bupivacaine liposome suspension, including an onset similar to lidocaine and duration of pain control similar to a combination of lidocaine and meloxicam (Martin et al., 2021). In the present study, a treatment effect was observed for the following outcomes: front stance time, front limb force, back position, and PGEM concentrations. Calves that received an analgesic showed increased front stance time, decreased front limb force, a less hunched back position, and lower PGEM concentrations, which collectively demonstrate a reduction in pain biomarkers.

Surgical castration seems to impact multiple outcomes captured by pressure mat gait analysis for both front and rear limbs. Kleinhenz et al. (2018) found that surgically castrated calves placed more force on their forelimbs compared with non-castrated controls, which is consistent with our findings from baseline values. Positive controls (castrated without analgesia) also had a decreased front stance time relative to calves administered an NSAID in the present study. Shortened front stance time may be indicative of calves being painful and warrants further investigation.

No significant treatment effects were observed for chute defense scores or pain scores. Relatively low chute defense scores and relatively high pain scores were observed across all treatment groups. The two scoring systems quantify different behaviors that, given the results of the current study, may not always agree. When individual pain behaviors were analyzed that comprised the pain scale adapted from Gleerup et al. (2015), differences were seen in the back position between the calves administered an NSAID and all other treatment groups not administered an NSAID. A previous study evaluating pain in Nellore cattle following castration found that restricted movement, such as an arched back, was more often observed in cattle experiencing pain (de Oliveira et al., 2014). In the present study, foot stamping seemed to increase immediately following castration which may have been due to lidocaine being irritating at the injection site, whereas attention to, and licking of, the surgical site did not increase until the 8 and 24 h time points, indicating that different pain behaviors may be more pronounced at different time points following surgical castration. Attention to, and licking of, the surgical site may have become more pronounced at 8 and 24 h due to these time points being beyond the local anesthetic duration of action (Riviere and Papich, 2018). Due to the diminishing anesthesia effects, the calves may be experiencing more localized pain. Ruminating was higher in the CON group relative to other treatments. Teeth grinding is a pain behavior described in Gleerup et al. (2015) that may have been quantified as ruminating in the ethogram used to score behavior and activity, further contributing to the idea that increased specificity in identifying behaviors may be valuable as opposed to combined pain and activity scoring. Some activities such as eating, standing, and walking were likely influenced by whether calves were fed during data collection. However, the −24 and 24 h time points were likely comparable and time spent walking at 24 h was less than at −24 h, indicating that activity may be reduced following castration. Failing to detect further differences in chute defense, pain and activity behaviors may have been due to a limited sample size and calves being partially or fully out of view of the camera on average 60 ± 4% of the time.

In the current study, the concentration of PGEMs only differed between treatments in which calves were treated with meloxicam relative to negative controls. These findings are consistent with previous findings, suggesting that NSAIDs reduce prostaglandin E_2_ concentrations over the duration of action of the drug (Stock et al., 2016). Concentrations in calves that did not receive an NSAID showed the greatest percent change from baseline at 4 h when the cortisol spike was beginning to decline and potentially was no longer suppressing aspects of the inflammatory response causing a rise in PGEM concentration (Van Engen and Coetzee, 2018).

Stewart et al. (2010) observed an immediate decrease in the ocular temperature following castration in calves that did not receive a local anesthetic, followed by an increase in the ocular temperature. Additionally, calves administered a local anesthetic exhibited increased ocular temperature immediately following castration (Stewart et al., 2010), which is consistent with the findings in the present study.

Cortisol concentrations were elevated immediately following castration and remained elevated for 24 h in CON calves and 4 h in treated calves, indicating that local anesthesia seemed to have an effect between 4 and 24 h on cortisol levels. At 24 h, calves in the BUP group had lower cortisol levels than the CON group indicating that while the duration of effect for lidocaine had likely ended, the bupivacaine liposome suspension may have still been having an effect. Roberts et al. (2015) did not find evidence of an effect of meloxicam on cortisol concentrations following castration which is similar to our findings. A meta-analysis of castration with and without analgesia found no significant differences in cortisol concentrations between surgically castrated calves not given analgesia and shams, and a tendency for analgesia to decrease cortisol levels after 120 min of intervention (Canozzi et al., 2017). Cortisol concentrations should be interpreted cautiously due to the stress of restraint during castration as well as blood sample collection.

Evidence from the current study indicates that surgical castration resulted in changes from baseline values in ocular temperature, pressure mat gait analysis, attention to and licking the surgical site, time spent standing and walking, and cortisol and PGEM concentrations. Previous research reports increased (Stewart et al., 2010) and decreased ocular temperature (Kleinhenz et al., 2018), increased force on front limbs (Kleinhenz et al., 2018), decreased overall activity and increased tail flicks (Laurence et al., 2016; Meléndez et al., 2018a), and increased cortisol concentrations (Kleinhenz et al., 2018; Meléndez et al., 2018b) following surgical castration. Significant changes from baseline values were still evident 120 h following castration, at the completion of the study, with increased ocular temperature, decreased gait distance, increased velocity, decreased front and rear stride length, decreased front and rear force, decreased front pressure, increased rear stance time, increased rear impulse, and increased PGEM concentrations. Previous studies have shown that pain associated with castration may persist up to 35 d after the procedure in 4- to 5-mo-old calves (Marti et al., 2017), which was well beyond the duration of the present study but demonstrates the need for extended duration analgesic regimens. The National Dairy FARM Animal Care Program encourages dairy calves to be castrated at as early an age as possible (National Milk Producers Federation, 2020). The American Association of Bovine Practitioners (AABP) Castration Guidelines state that castration should be performed as early as possible from 1 d to 3 mo of age but that age of castration may vary between production systems and should be based upon veterinarian recommendations (AABP, 2019).

No drugs are currently labeled to control pain associated with castration in cattle in the United States; thus, extra-label drug use under the AMDUCA is the only way in which this use is permitted (FDA, 1994). In a recent survey, both producers and veterinarians selected “I am not comfortable using an analgesic unless it has been approved by the FDA” as a common reason for not using an analgesic (Robles et al., 2021). Producers’ ability to implement a change is key for adopting new means of pain control (Jansen et al., 2009). Currently, bupivacaine liposome suspension is not readily available or likely cost-effective for producers to implement. However, a recent survey suggests that analgesic use at the time of castration may be more common than it was 10 yr ago (Johnstone et al., 2021), and the cost, availability, and approval of analgesics may evolve over time, providing more options for pain control at the time of castration.

## Conclusions

Evidence provided in the current study demonstrates that pain from surgical castration can last up to 120 h post-castration, indicated by changes in ocular temperature, gait analysis, and PGEM concentrations. These data show that the administration of bupivacaine liposome suspension as a local anesthetic block at the time of castration was as effective at controlling pain as a multimodal approach of lidocaine and meloxicam. A single injection that alleviates both perioperative and postoperative pain would be an attractive option for livestock producers to alleviate pain at the time of castration. Further research is needed to discover effective ways of managing pain for extended durations following painful husbandry procedures.

## Abbreviations

BUP: bupivacaine
CON: control
IRT: infrared thermography
LID: lidocaine
MEL: meloxicam
NSAID: non-steroidal anti-inflammatory drug
PGEM: prostaglandin E_2_ metabolite
